# 

**Published:** 1882-05

**Authors:** 


CONTENTS.

How to Exercise .......... 225
It Will Out .......... 227
Beauty's Tyranny .......... 228
The Management of Sick Children .......... 231
Prevention of Disease .......... 231
A Story of Abraham Lincoln .......... 232
India Rubber Hunting .......... 233
The Dining Hall in the Thirteenth Century .......... 235
Wild Animals and their Tamers .......... 236
English as the Speech of the Future .......... 237
A Machine that Measures Thought .......... 238
Dangers of Spring .......... 241
Mind and Health .......... 243
Hardships of Farmers' Wives .......... 245
Ignorance and Ill Health .......... 249
Rheumatism .......... 250
Sorrowing Poverty .......... 251
Traveling for Health .......... 252
The Family Relation .......... 259
How to Save a Drowning Person .......... 261
Reflections upon the Life of Medical Men .......... 262
Paragraphs .......... 265
Shaving the Beard .......... 271
Reason in All Things .......... 272
Killing a Rhinoceros .......... 273
Unconscious Reading .......... 274
Chinese Pirates .......... 275
An Anecdote of Waterloo .......... 278
A Literary Curiosity .......... 280
"Uncle Sam" .......... 282

